# RNA Interference against Discoidin Domain Receptor 2 Ameliorates Alcoholic Liver Disease in Rats

**DOI:** 10.1371/journal.pone.0055860

**Published:** 2013-02-07

**Authors:** Zheng Luo, Huimin Liu, Xiaomeng Sun, Rong Guo, Ruibing Cui, Xiangxing Ma, Ming Yan

**Affiliations:** 1 Department of Geriatric Gastroenterology, Qilu Hospital of Shandong University, Jinan, Shandong, China; 2 Key Laboratory of Cardiovascular Remodeling, Qilu Hospital of Shandong University, Jinan, Shandong, China; 3 Department of Hepatology and Gastroenterology of Yantai Yuhuangding Hospital of Qingdao University Medical College, Yantai, Shandong, China; 4 Department of Radiology, Qilu Hospital of Shandong University, Jinan, Shandong, China; National Institutes of Health, United States of America

## Abstract

Discoidin domain receptor 2 (DDR2) is involved in fibrotic disease. However, the exact pathogenic implications of the receptor in early alcoholic liver disease are still controversial. We constructed plasmid vectors encoding short-hairpin RNA against DDR2 to investigate its role in alcoholic liver disease in an immortalized rat hepatic stellate cell line, HSC-T6, and in rats by MTT, RT-PCR and western blot analyses; immunohistochemistry and electron microscopy. Alcohol-induced upregulation of DDR2 was associated with the expression of matrix metalloproteinase 2, the transforming growth factor β1 signaling pathway and tissue inhibitor of metalloproteinase 1; collagen deposition; and extracellular matrix remodeling. Inhibition of DDR2 decreased HSC-T6 cell proliferation and liver injury in rats with 10-week-induced alcoholic liver disease. DDR2 may have an important role in the pathogenesis of early-stage alcoholic liver disease. Silencing DDR2 may be effective in preventing early-stage alcoholic liver disease.

## Introduction

Alcoholic liver disease (ALD) is a major cause of morbidity and mortality. It begins with the fatty liver and proceeds to liver inflammation, necrosis, progressive fibrosis and hepatocellular carcinoma. The stage before fibrosis can be cured if discovered in time and treated properly [1]. Histopathologic features of ALD include sinusoidal capillarization, deposition of collagen, and ballooning degeneration of hepatocytes [2]. Sinusoidal capillarization is characterized by loss of differentiation of liver sinusoidal endothelial cells and the formation of basement membrane in the Disse space. It impedes the metabolic exchange of nutrients and waste and precedes the onset of hepatic fibrosis. The formation of basement membrane results from increased sinusoidal deposition of laminin and collagen produced by activated hepatic stellate cells (HSCs) [3]. Ballooning degeneration of hepatocytes is a form of cell swelling, enlargement, rounding and reticulated cytoplasm [4]. The biochemical indexes of serum aspartate aminotransferase (AST), serum alanine aminotransferase (ALT), especially their ratio suggest the degree of liver injury, and the ratio of AST/ALT indicates the degree of ALD [5]. Ethanol could increase liver weight, and cause losing appetite, nausea, at last body weight decreased. So the ratio of liver to body weight in ALD was increased [6], [7], [8].

HSCs, located in the perisinusoidal spaces, a small area between the sinusoids and hepatocytes, are the major effectors of ALD. During the early stages of hepatic injury, the normally quiescent, vitamin-A-storing HSCs transform into actively proliferating, collagen-producing myofibroblast-like cells [9]. Activated HSCs demonstrate a plastic and metabolitically active phenotype that is proliferative and fibrogenic, during resolution of liver injury, activated HSCs have been demonstrated to undergo apoptosis [10]. Acetaldehyde, a metabolite of alcohol, is associated with the activation and perpetuation of HSCs by increasing collagen I content [11], [12]. HSCs participate in extracellular matrix (ECM) remodelling by producing transforming growth factor beta β1 (TGF-β1), tissue inhibitor of metalloproteinase 1 (TIMP-1), α-smooth muscle actin (α-SMA), collagen and matrix metalloproteinases (MMPs, especially MMP2), which degrades the normal subendothelial ECM [13], [14]. TGF-β1 has an important role as a profibrogenic factor in chronic liver disease, triggering the expression of TIMP-1, a key effector of fibrogenesis [15], [16]. ECM is a dynamic regulator of cell function, whose degradation hastens its replacement by fibril-forming collagen and the formation of basement membrane, then further HSC proliferation and MMP2 production occur in a positive feedback loop [17].

The interaction of collagen and HSCs may be mediated by tyrosine kinase receptor-like discoidin domain receptor 2 (DDR2) [18]. DDR2 is expressed in some tumor cells and some fibrotic diseases of the heart, lung, kidney, cartilage, skin and liver [19], [20], [21]. DDR2 expression induced in liver fibrosis models was detected in activated HSCs but not hepatocytes or Kupffer cells [22], [23], [24]. DDR2 is characterized by 3 distinct regions: an extracellular domain for collagen binding, a transmembrane region and an intracellular kinase domain [25]. Binding of collagen(s) to the extracellular domain could induce tyrosine phosphorylation of the kinase domain [26]. Prolonged activation of the DDR2 kinase domain results in upregulation or activation of MMP2 linked to the control and neo-synthesis of ECM molecules. MMP2-mediated proliferation and invasion of HSCs can aggravate ALD [18], [27], [28]. However, DDR2, HSCs and macrophages may combine to attenuate chronic hepatic fibrosis [29], [30]. So the exact role of DDR2 in ALD remains unknown.

Targeted posttranscriptional gene silencing by RNA interference (RNAi) can inhibit gene expression *in vitro* and *in vivo*. Transduction of naked plasmid with short hairpin RNA (shRNA) is a safe and effective method of gene delivery. Rapid intravenous injection of physiological buffer in a large volume can achieve effective localization mainly in the liver [31].

In this study, we used shRNA of DDR2 to investigate its role in HSC activation, proliferation, necrosis and apoptosis, sinusoidal capillarization and collagen deposition *in vitro* and *in vivo* in rats with early stage ALD.

## Materials and Methods

### Cell Culture and Transfection

HSC-T6 cells, an immortalized rat HSC cell line [32] with an activated HSC phenotype, were obtained from the Institute of Basic Medical Sciences of Qilu Hospital (originally from Liver Disease Research Center of San Francisco General Hospital, CA, USA). Cells were cultured in DMEM supplemented with 10% fetal bovine serum (FBS) (Gibco, New Zealand, USA) under a humidified atmosphere of 5% CO_2_ at 37°C in 60-mm flasks. When cells reached 90% confluence, they were transfected with the plasmid PGPU6/GFP/Neo-shRNA targeting DDR2 (p.DDR2.shRNA; 8.0 µg with 10 µl Lipofectamine 2000 [Invitrogen, Shanghai] in each flask). The medium was changed after 6 h. The control group received 10 µl Lipofectamine alone. The efficiency of transfection was observed by fluorescence microscopy (Olympus IX-70; Olympus, Prague). Then cells were cultured in DMEM with 10% FBS and 500 µg/ml G418 (Invitrogen, Shanghai) for 2 weeks, and monoclonal cells were cultured for 1 week. Before stimulation, cells were serum starved for 48 h in DMEM with 0.4% FBS to minimize the formation of adducts between acetaldehyde and serum proteins [33]. Acetaldehyde was added (100 µM) for 24 or 48 h. The acetaldehyde medium was replaced every 12 h.

### Screening for siRNA

Three short interfering RNAs (siRNAs) were transfected (20 pmol with 1 µl Lipofectamine) into stimulated HSC-T6 cells (acetaldehyde 100 µM/ml for 24 h) and the effective siRNA was selected by PCR for further research. Primer sequences were for Ddr2-s1 5′-AGAGAGUGCUACCAAUGGU(dTdT)-3′; Ddr2-s2, 5′-CGACUCUGUGUAUAAGCUG(dTdT)-3′; Ddr2-s3, 5′-AGAGUAACCCUUAUGAUGU(dTdT)-3′; and one scramble sequence, 5′-UUCUCCGAACGUUCACGU(dTdT)-3′ as a negative control. These sequences were submitted to a BLAST search of the NCBI database to ensure that only the DDR2 gene was targeted. The effective siRNA and scramble sequence were combined with PGPU6/GFP/Neo-shRNA expression vectors (p.DDR2.shRNA and p.control.shRNA) (Gene Pharma, Shanghai).

### Quantitative Real-time PCR Analysis

Total RNA was homogenized and extracted from cells and frozen specimens by use of Trizol (Takara, Shiga, Japan). The RNA samples underwent reverse transcription with M-MLV reverse transcriptase (Invitrogen, Shanghai) and random primers. The resulting cDNAs (1 µg) were amplified with Taq DNA polymerase (Invitrogen, Shanghai) with 35 PCR cycles of 2 min at 94°C, then 94°C for 30 s, 59°C for 30 s and 72°C for 30 s, and then 72°C for 5 min. Primer sequences are in Table 1. Primers were synthesized by BioAsia Corp. (Shanghai). DDR2 PCR products were visualized by UV light on 1.5% agorose gel. The mRNA expression of transforming growth factor-β1 (TGF-β1), tissue inhibitors of metalloproteinase 1 (TIMP-1), and collagen I was quantitatively determined on a Light Cycler apparatus (Roche Diagnostics, Mannheim, Germany) with use of SYBR green Master Mix (Takara, Japan). The primers were designed by use of Primer Premier 5.0 (Premier Biosoft International, Palo Alto, CA, USA) with mRNA sequences from GenBank. The fold increase compared with control cells was determined by the 2^−ΔΔCT^ method (Schmittgen and Livak, 2008) with β-actin as a normalizing gene.

**Table 1:** GenBank accession numbers and sequences for primers.

Target gene	GenBank accession no.	Primer	Nucleotide sequence
DDR2	NM 031764.3	Forward	5′-AGCGAGTCCAGCATGTTCAATAACA-3′
		Reverse	5′-GGTAGTCAGGGCGAAGGGGAA-3′
TIMP-1	NM_053819.1	Forward	5′-ATTTGCACATCACTGCCTGC-3′
		Reverse	5′-GGGATGGCTGAACAGGGAAA-3′
TGF-β1	NM_021578.2	Forward	5′-CTGCTGACCCCCACTGATAC-3′
		Reverse	5′-GTGAGCACTGAAGCGAAAGC-3′
collagen I	NM_053304.1	Forward	5′-GACTGTCCCAACCCCCAAAA-3′
		Reverse	5′-CTTGGGTCCCTCGACTCCTA-3′
actin	NM_031144.2	Forward	5′-CGTTGACATCCGTAAAGACC-3′
		Reverse	5′-TAGAGCCACCAATCCACACA-3′

### Western Blot Analysis

Cells and tissue were lysed in RIPA buffer (Beyotime, Shanghai) of 50 mM Tris (pH 7.4), 150 mM NaCl, 1% Triton X-100, 1% sodium deoxycholate, 0.1% SDS and protease inhibitor cocktail with 1 mM phenylmethanesulfonyl fluoride (Beyotime). Protein (20 µg) was separated by SDS-PAGE and transferred to PVDF membranes (Millipore, Bedford, MA, USA) that were blocked and incubated with the primary antibodies against DDR2 (1∶800), MMP2 (1∶1000; both Abcam, MA, USA), p-Smad2/3 (1∶500) and actin (1∶3000; both Santa Cruz Biotechnology, CA, USA) at 4°C overnight, then horseradish peroxidase-conjugated goat anti-rabbit IgG or donkey anti-goat IgG (1∶5000; Santa Cruz Biotechnology, CA, USA) at room temperature for 2 h. The film was analyzed by use of an image analyzer (Alpha Innotech, San Leandro, CA, USA) with the Immobilon Western Chemiluminescent HRP Substrate kit (Millipore, Billerica, MA, USA). Western blot analysis was quantified with use of Quantity One software (Bio-Rad, USA).

### MTT Assay

MTT assay were used to determine cell proliferation. Cells were seeded in 96-well plates at 1×10^5^/wells and incubated in 200 µl culture medium. A 20-µl stock MTT solution (Promega, Madison, WI, USA) (5 mg/mL) was added to each well for further incubation for 4 h at 37°C. The medium was carefully removed, then 200 µl dimethyl sulfoxide was added to each well for vibrating for 10 min. The absorbance at 570 nm was measured by use of an automated plate reader.

### Hoechst 33342 and PI Staining

To determine apoptotic and necrotic nucleus changes, cells were seeded in 12-well plates at 1×10^6^/wells and incubated in 1 ml culture per well. Cells were stained with Hoechst 33342 (10 µl) and PI (5 µl) solution (KeyGen Biotech, Nanjing, China) in the dark for 10 min each, then viewed under a fluorescence microscope (Olympus IX-70; Olympus, Prague). The results were analyzed by use of Image-Pro Plus 6.0 (Media Cybernetics, Bethesda, MD, USA).

### Rat Model of ALD

This work was approved by the institutional review board of Qilu Hospital. We obtained 60 male Sprague-Dawley rats (6 weeks old) from the Experimental Animal Center of Shandong University. Rats were randomly divided into 5 groups (n = 12 each): control, sham, ALD, p.DDR2.shRNA+ALD and p.control.shRNA+ALD. Each group was fed standard food with olive oil. The normal control group received no intragastrical or intravenous treatment. For alcohol treated groups, an amount of 60% alcohol (Red Star Er Guo Tou Jiu, Beijing) was diluted with water and given intragastrically in increasing doses: 4.5, 6.5 and 9 g/kg/day for 1–4, 5–8, and 9–10 weeks, respectively. The daily dose of alcohol was administered in 2 divided doses, 12 h apart. The sham group received dextrose to isocalorically replace alcohol. Rats were weighed at the beginning and end of the experiment; livers were weighed at the end of the experiment, blood samples were obtained from left ventricles, and rats were killed by intraperitoneal injection of sodium pentobarbital to the right lower abdomen. Animal welfare and experimental procedures were in accordance with the care and use of laboratory animals (National Research Council, Washington, DC, USA).

### Gene Delivery to Rats

From week 2 of alcohol ingestion, rats received rapid intravenous tail-vein injections of liposome-encapsulated plasmids, with 1∶0.5 ratio of DNA (µg) to Lipofectamine (µl), and saline water for the sham group (in a volume of DMEM not exceeding 1 ml). Rats received 0.2 mg/kg body weight plasmid twice a week for 8 weeks.

### Histochemistry

Liver lobes were trimmed from the porta hepatis and weighed; liver tissue was harvested from the right hepatic lobe and fixed with 4% paraformaldehyde solution. Paraffin-embedded liver sections were stained with hematoxylin and eosin (H&E), Masson's trichrome or Gordon and Sweet's reticulin stain for light microscopy, or Picrosirius red for polarization microscopy. Other sections were incubated with a citrate-buffer microwave repair antigen and 3% H_2_O_2_ at 25°C for 10 min, washed 3 times with PBS, then incubated in a humidified chamber with serum (Zhongshan Golden Bridge Biotechnology Co., Beijing) for 15 min at 25°C. Antibodies for DDR2 (1∶100), MMP2 (1∶500), laminin (1∶100), α-SMA (1∶100), (Abcam, MA, USA) and p-Smad2/3 (1∶100) were added for incubation at 4°C overnight. A streptavidin peroxidase immunohistochemistry kit was used (Zhongshan Golden Bridge Biotechnology Co.). Immune peroxidase activity was developed in DAB for the indicated times. Hepatic tissue sections were observed under an optical microscope (Olympus BX51; Olympus, Tokyo). The results were analyzed by use of Image-Pro Plus 6.0.

### Electron Microscopy of Hepatic Tissue

Tissue samples of the right hepatic lobe were washed rapidly in phosphate buffer and fixed in 3% glutaraldehyde. After 2–3 min, samples were cut into 1-mm^3^ pieces and fresh 3% glutaraldehyde was added. After a rinsing, samples were fixed in OsO4, rinsed and dehydrated, then embedded in Epon 812 epoxy resin before ultrathin sections were cut. Sections were stained with lead citrate and uranyl acetate. Ultrastructures of sections were visualized by use of a JEM-1200EX electron microscope (JEOL Co., Tokyo).

### Measurement of Apoptosis in Paraffin Tissue Sections

We used the TdT-mediated dUPT nick end-labeling (TUNEL) method (Roche, Mannheim, Germany) to examine the apoptosis rate in activated HSCs. Tissues were deparaffinized with xylene and rehydrated with graded dilutions of ethanol and 2 washes in PBS. The tissue sections were digested with proteinase K (20 µg/ml in Tris-HCl (Beyotime, Jiangsu, China) for 30 min at 37°C. The slides were washed 3 times in PBS and incubated with 50 µl of the TUNEL reaction mixture (TdT and fluorescein-labeled dUTP) in a humid atmosphere for 60 min at 37°C. After 3 washes in PBS, the sections were incubated for 30 min with Converter-POD. The sections were visualized with DAB substrate, with nuclei with DNA fragmentation stained brown. The reaction was terminated after the indicated times with PBS. To determine the proportion of brown-stained apoptotic nuclei of HSCs, tissue was counterstained with a polyclonal antibody specific for α-SMA. Endogenous peroxidase was blocked with a 3% H_2_O_2_ solution in PBS for 15 min, then washed with 20% goat serum solution. The antibody was incubated overnight (1∶100) at 4°C. After 3 washes, sections were treated with anti-rabbit IgG and then biotin-conjugated secondary antibody for 30 min (Zhongshan Golden Bridge Biotechnology Co.). An avidin-biotin complex was added for an additional 30 min, and HSC cytoplasm was visualized after 1 min of exposure in DAB substrate. Then sections were stained with hematoxylin. Apoptotic HSCs were stained brown in both nuclei and cytoplasm and other activated HSCs were stained brown in cytoplasm but blue in nuclei. Apoptotic HSCs were quantified by use of Image-Pro Plus 6.0. Apoptosis rate was expressed as ratio of TUNEL-positive HSCs to total activated HSCs per 10^3^µm^2^ of liver tissue [34].

### Immunohistochemistry of Ki-67 Expression

To obtain the proliferation ratio in activated HSCs, paraffin-embedded sections were microwaved in a citrate buffer and stained with a monoclonal rat antibody with specificity against rat Ki-67 at 1∶100 dilution (eBioscience, San Diego, CA) and incubated overnight at 4°C. After a wash, sections were treated by use of the streptavidin peroxidase immunohistochemistry kit described above. Immune peroxidase activity was developed in DAB for approximately 1 min, and positive-stained nuclei were brown. Sections were then incubated overnight at 4°C with a polyclonal antibody against α-SMA, and positive-stained cell cytoplasm was brown. Then sections were stained with hematoxylin. Proliferative HSCs were stained brown in both nuclei and cytoplasm and other activated HSCs were stained brown in cytoplasm but blue in nuclei. Proliferating cells were quantified by use of Image-Pro Plus 6.0. Proliferation rate was expressed as ratio of ki67-positive HSCs to total activated HSCs per 10^3^µm^2^ of liver tissue.

### Biochemistry

Rat blood was allowed to stand for 10 min before being centrifuged at 2,000 rpm for 10 min to obtain serum. The serum levels of ALT and AST were assayed at the clinical laboratory of Qilu Hospital.

### Statistical Analysis

Data are expressed as mean ± SD. Analysis involved one-way ANOVA followed by Tukey’s post-hoc test for more than 2 groups with use of SPSS v17.0 (SPSS, Inc., Chicago, IL, USA). P<0.05 was considered statistically significant.

## Results

### Characterization of Effective Anti-DDR2 siRNA

**Figure 1:**
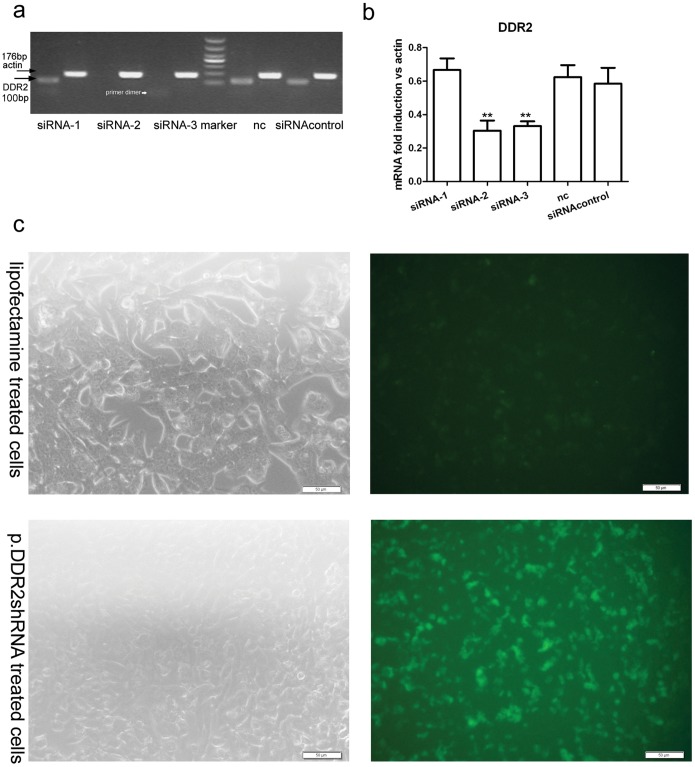
Screening of discoidin domain receptor 2 (DDR2) siRNA and transfection efficiency of short hairpin RNA plasmid (p.DDR2.shRNA). (a) RT-PCR analysis of mRNA level of DDR2 in siRNA-transfected immortalized rat hepatic stellate cell line HSC-T6. Arrows indicate the position of DDR2 and actin. (b) Quantification of mRNA level of DDR2 relative to that of actin. Data are mean ± SD (n = 3). **P<0.01 *vs* normal and siRNA control. (c) Light (left) and fluorescence microscopy (right) of transfection efficiency of p.DDR2.shRNA as compared with no plasmid.

Among all 3 siRNAs tested, siRNA-2 and siRNA-3 were potent DDR2 silencers as compared with the normal control, but siRNA-3 contained a primer dimmer. Scramble siRNA of DDR2 did not affect DDR2 expression as compared with the normal control (**Fig. 1a,b**). DDR2 siRNA-2 was chosen as an effective DDR2 siRNA.

### Efficient Transfer of p.DDR2.shRNA into HSC-T6 Cells and Rats

**Figure 2:**
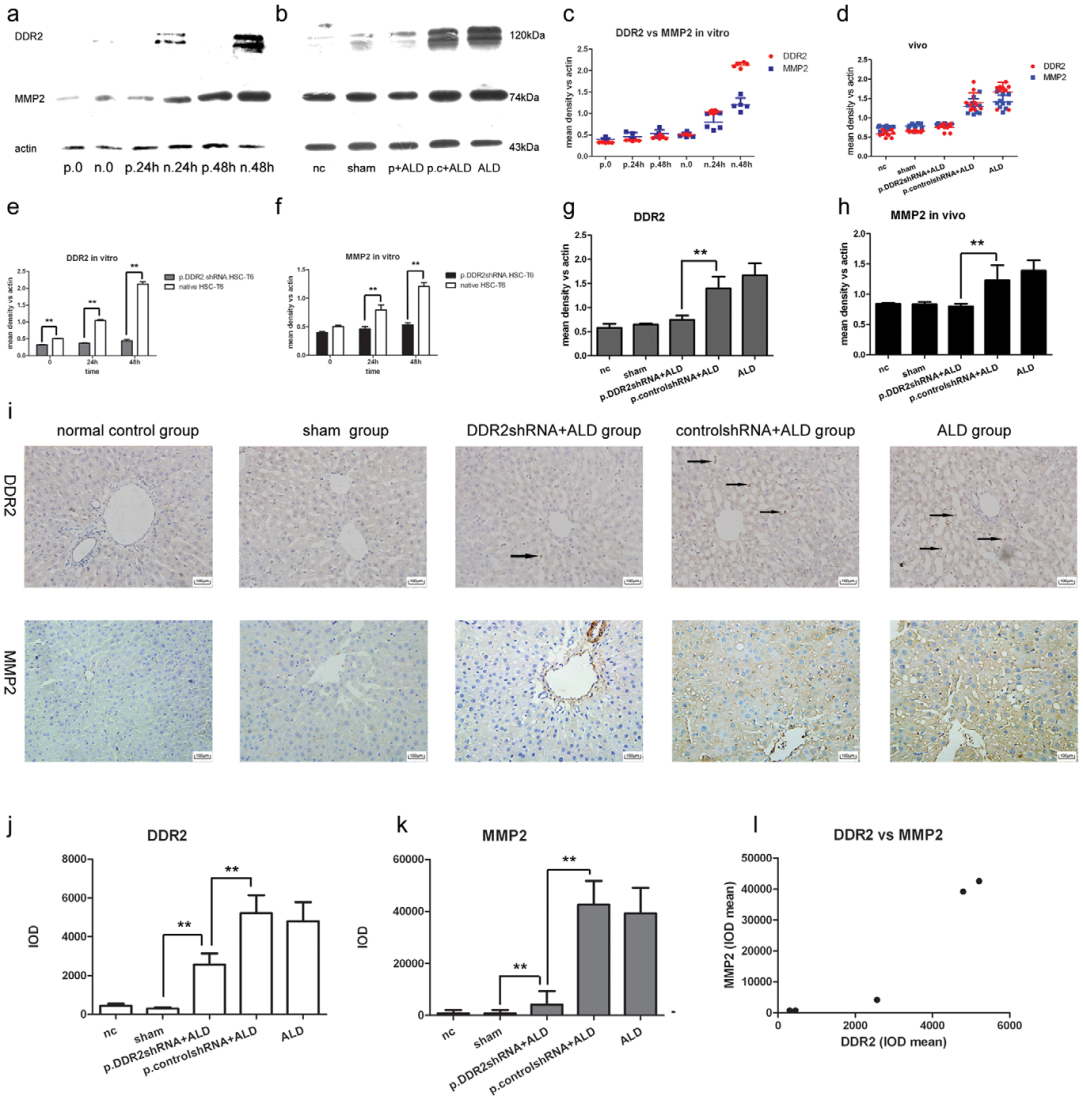
Association of DDR2 and matrix metalloproteinase 2 (MMP2) levels in alcoholic liver disease (ALD). (a) Western blot analysis of protein level of DDR2 in HSC-T6 cells with acetaldehyde treatment for the indicated times (n = 3). 0, cells without acetaldehyde treatment. (b) Western blot analysis of protein levels of DDR2 and MMP2 in rat liver tissue with normal control, sham, ALD, p.control.shRNA+ALD and p.DDR2.shRNA+ALD treatment (n = 10) and (c–h) quantification relative to that of actin (n = 3–20). (i) Immunohistochemistry of levels of DDR2 and MMP2 in rat liver. 400×magnification. Arrows show DDR2-positive HSCs. (j–l) Quantification of immunohistochemistry results (n = 20). Data are mean ± SD. *P<0.05, **P<0.01.

The GFP-expressing plasmid p.DDR2.shRNA was transfected into HSC-T6 cells, and the efficiency of transduction was approximately 60% (**Fig. 1c**). We stimulated native and p.DDR2.shRNA-treated HSC-T6 cells with acetaldehyde (100 µM/L) for 24 or 48 h. p.DDR2.shRNA significantly inhibited the protein expression of DDR2 (**Fig. 2a,c**). *In vivo*, gene therapy inhibited the protein expression of DDR2 (**Figs. 2b,d**). Immunohistochemistry demonstrated that positive staining of DDR2 in perisinusoidal spaces was less with gene therapy than with ALD treatment (**Fig. 2i–k**).

### Acetaldehyde Upregulated and DDR2 shRNA Decreased DDR2 and MMP2 Protein Levels

Acetaldehyde increased the protein level of DDR2 and MMP2 *in vitro* and *in vivo* (**Fig. 2a–h**). Immunochemical staining demonstrated DDR2-positive labeling on HSCs and MMP2-positive labeling in liver in alcohol treated group (**Fig. 2i–l**). The protein level of DDR2 and MMP2 was lower with than without p.DDR2.shRNA in HSC-T6 cells, and lower in the ALD group but higher than in the sham group (**Fig. 2a, g and h**). Protein levels did not differ between ALD and p.control.shRNA+ALD groups or normal control and sham groups.

### Acetaldehyde Upregulated and DDR2 shRNA Decreased TIMP-1 and Collagen I mRNA Levels and Changed the TGF-β1 Signal Pathway

**Figure 3:**
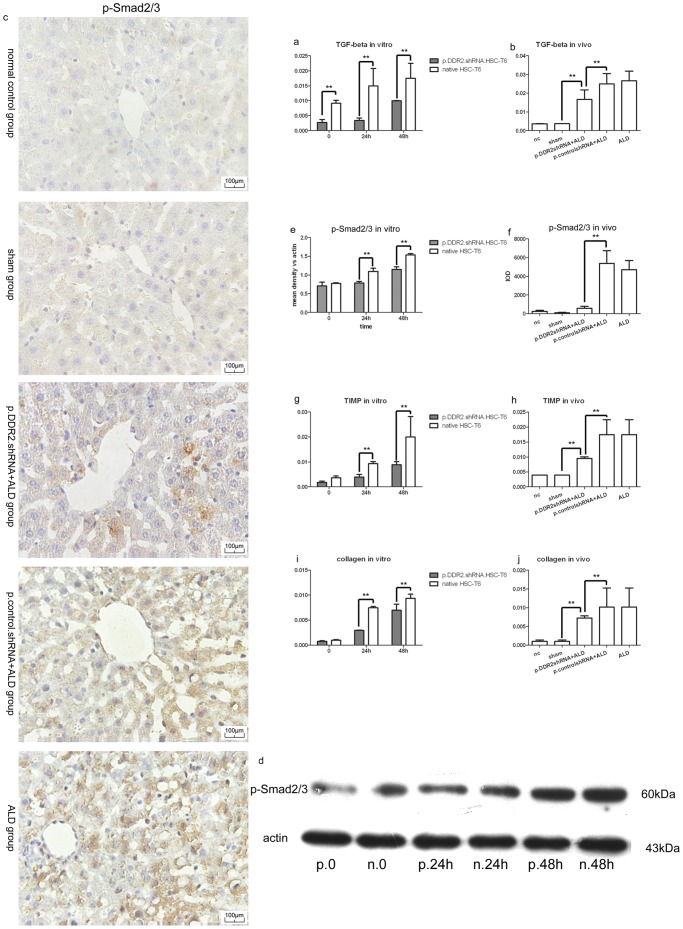
Knockdown of DDR2 inhibited transforming growth factor β1 (TGF-β1) signaling pathway, tissue inhibitor of metalloproteinase 1 (TIMP1) and collagen I mRNA expression. (a) Quantification of mRNA level of transforming growth factor β1 (TGF-β1) in HSC-T6 cells (n = 4) (0, cells without acetaldehyde stimulation treatment) and (b) rat liver tissue (n = 10). (c) Immunohistochemistry of p-Smad2/3 levels in rat liver. 400× magnification. (d) Western blot analysis of protein level of p-Smad2/3 in HSC-T6 cells with acetaldehyde treatment for the indicated times (n = 3). 0, cells without acetaldehyde treatment. Quantification of p-Smad2/3 levels by immunohistochemistry and western blot analysis (n = 3–20) (e,f). Quantification of mRNA level of TIMP1 in HSC-T6 cells (n = 4) (g) and rat liver tissue (n = 10) (h). Quantification of mRNA level of collagen I in HSC-T6 cells (n = 4) (i) and rat liver tissue (n = 10) (j). Data are mean ± SD. **P<0.01.

Acetaldehyde stimulated the expression of TGF-β1 and p-Smad2/3 and increased the mRNA levels of TIMP-1 and collagen I (**Fig. 3**). *In vitro* and *in vivo*, the level of TGF-β1 mRNA (**Fig. 3a,b**) and protein expression of p-Smad2/3 *in vitro* (**Fig. 3d**) and *in vivo* (**Fig. 3c**) were lower with DDR2 shRNA treatment than acetaldehyde (for cells) or ALD (for rats) treatment (**Fig. 3a–f**). The mRNA levels of TIMP-1 and collagen I were lower with than without p.DDR2.shRNA in cells and lower in the p.DDR2.shRNA than ALD group but higher than in the sham group (**Fig. 3g–j**). The levels did not differ between ALD and p.control.shRNA+ALD groups or normal control and sham groups.

### DDR2 shRNA Increased the Necrosis Rate and Reduced Proliferation Rate of HSCs and Altered Rat Liver and Body Features

**Figure 4:**
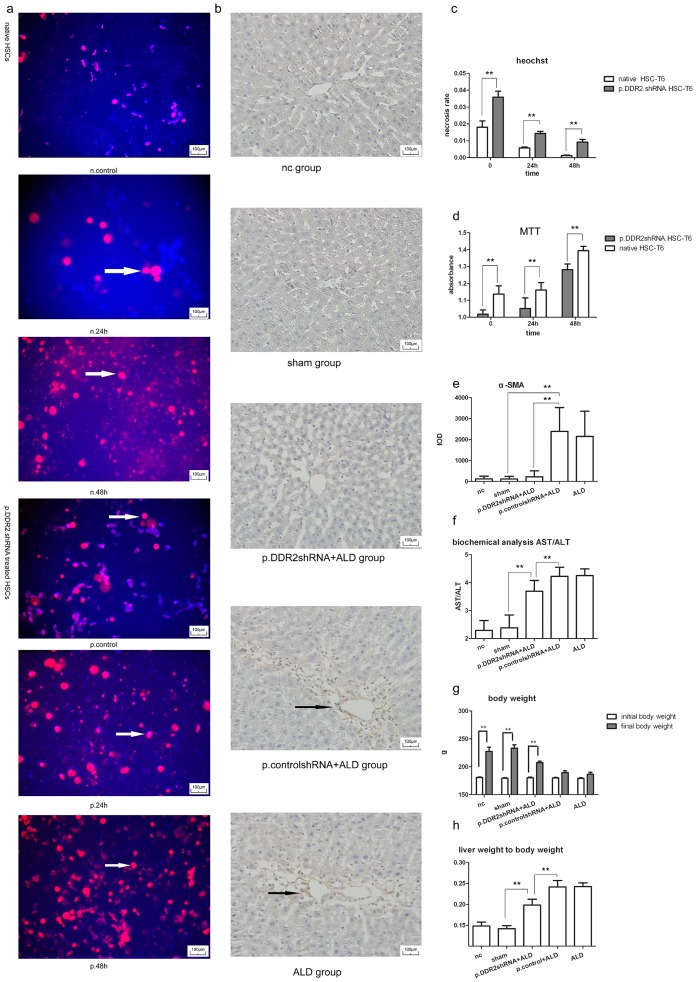
Knockdown of DDR2 inhibited proliferation, activation and induced necrosis in HSCs and changed the body features of rats. (a) Hoechst 33342 and PI staining of necrosis of HSC-T6 cells with acetaldehyde treatment for the indicated times. Live cells show a low level of fluorescence; apoptotic cells show a higher level of blue fluorescence, and necrotic cells show low-blue and high-red fluorescence. Arrows show necrotic cells. (b) Immunohistochemical staining for α-SMA in liver tissue (400×magnification); arrows show positive stained cells. (c) Quantification of Hoechst 33342 and PI staining. 0, cells without acetaldehyde treatment (n = 5). (d) Quantification of MTT results (n = 8). (e) Quantification of immunohistochemical staining for α-SMA (n = 20). (f) Quantification of biochemistry (n = 10). (g) Body weight and (h) liver weight to body weight (n = 10 each). Data are mean ± SD. *P<0.05, **P<0.01.

Hoechst 33342 and PI staining revealed significantly more necrosis in p.DDR2.shRNA-treated than native HSC-T6 cells. Acetaldehyde induced cell necrosis by stimulation time. Cells showed little Hoechst 33342 staining from 24 to 48 h (**Fig. 4a,c**).

We examined the effect of p.DDR2.shRNA on HSC activation during alcoholic liver injury by immunohistochemical staining of α-SMA. The number of activated HSCs, which express α-SMA and are therefore considered myofibroblast-like cells, was significantly increased in the ALD group, and p.DDR2.shRNA greatly reduced the number. Number of activated cells did not differ among normal control, sham and p.DDR2.shRNA+ALD groups or ALD and p.control.shRNA+ALD groups (**Fig. 4b,e**).

On MTT assay, cell proliferation was higher with than without acetaldehyde stimulation and lower with than without p.DDR2.shRNA treatment(**Table 2** **,** **Fig. 4d**).

**Table 2:** Proliferation of rat hepatic stellate cell line HSC-T6 with acetaldehyde treatment (100 µM/L) at 24 and 48 h.

	Untreated	24 h	48 h
Native HSC-T6 cells	1.14±0.05	1.16±0.04	1.39±0.03^b^
p.DDR2.shRNA-treated HSC-T6 cells	1.02±0.03^a^	1.05±0.06^a^	1.28±0.03^ab^

Data are mean ± SD, n = 8.

a P<0.01 compared with native HSC-T6 cells.

b P<0.01 compared with 24 h acetaldehyde-treated cells or untreated cells.

ALT, AST activities and the ratio of AST to ALT were higher in rats with ALD than the normal control; the ALT level has no significant differences between p.DDR2.shRNA and ALD group, but the AST level and the ratio of AST to ALT were significantly higher for the p.DDR2.shRNA than normal group but lower than the ALD group (**Table 3** **,** **Fig. 4f**). The results did not differ between normal control and sham groups or ALD and p.control.shRNA+ALD groups.

**Table 3:** Levels of alanine aminotransferase (ALT) and aspartate aminotransferase (AST), and initial and final body weight of rats.

	Normal control	Sham	p.DDR2.shRNA +ALD	p.control.shRNA +ALD	ALD
AST (U/L)	102.70±8.82	101.30±9.80	254.7±16.75^ab^	295.5±6.65^a^	297.4±12.3^a^
ALT (U/L)	45.4±5.44	43.3±6.04	74±4.99^a^	72.2±5.31^a^	70.2±6.65^a^
AST/ALT	2.29±0.36	2.39±0.46	3.45±0.22^ab^	4.11±0.29^a^	4.256±0.24^a^
Initial body weight (g)	180.8±3.55	179.6±2.95	180.4±3.98	180±3.40	179.4±3.53
Final body weight (g)	227.6±24.05^d^	233.4±18.60^d^	207.6±8.21^d^	189.6±10.66	186.8±11.20
Liver weight (g)	34.16±4.35	33.37±1.98	41.25±3.66^ac^	45.37±2.90^a^	45.19±3.21^a^
Liver weight/body weight(%)	14.82±0.94	14.19±0.72	19.83±1.38^ab^	24.14±1.53^a^	24.26±0.87^a^

Data are mean ± SD, n = 10.

a P<0.01 compared with normal control.

b P<0.05 compared with ALD.

c P<0.05 compared with ALD.

d P<0.01 compared with initial body weight.

After 10-week ingestion, final body weight was heavier than the initial body weight for p.DDR2.shRNA, normal control and sham groups but did not differ between ALD and p.control.shRNA+ALD groups. The ratio of liver weight to body weight was higher for the p.DDR2.shRNA than normal control group but lower than the ALD group. Body weight did not differ between normal control and sham groups or ALD and p.control.shRNA+ALD groups (**Table 3** **,** **Fig. 4g,h**).

### Knockdown of DDR2 Increased HSC Apoptosis and Decreased HSC Proliferation *in vivo*

**Figure 5:**
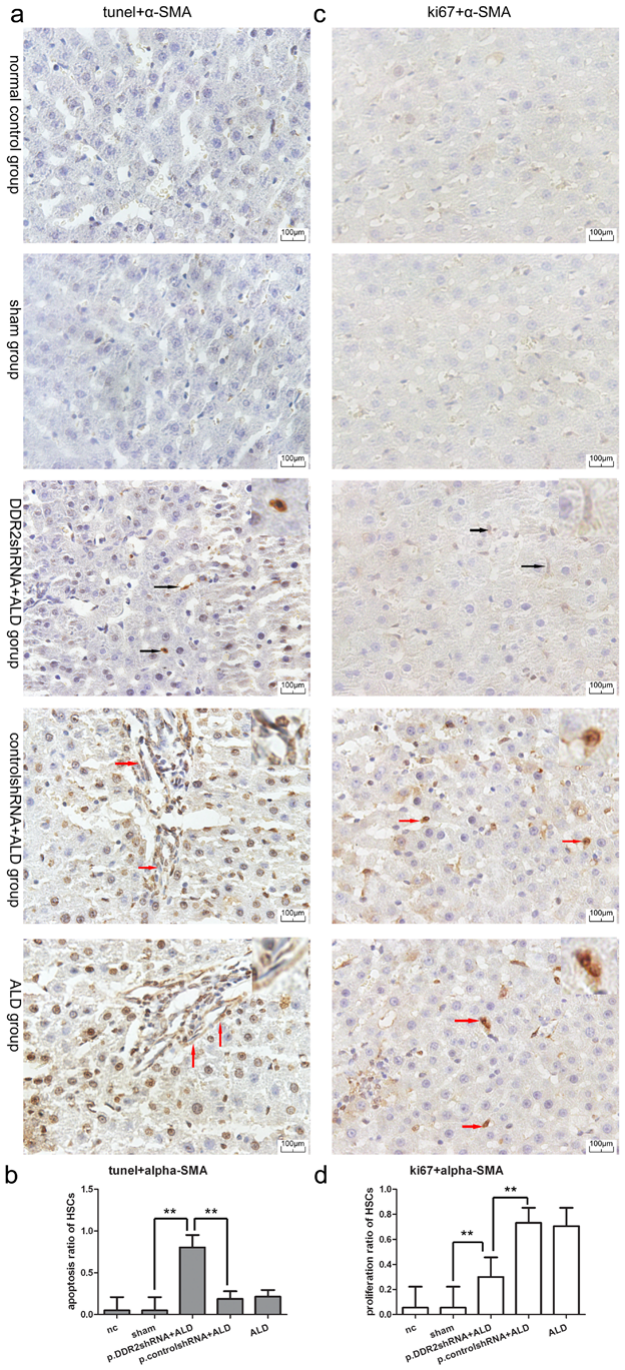
Knockdown of DDR2 induced apoptosis and inhibited proliferation of HSCs in rat liver. (a) Light microscopy of TUNEL and α-SMA dural stain (400×magnification); black arrows show positive cells with brown stain in both nuclei and cytoplasm, one of these cells was amplified at the upper right corner of image of p.DDR2.shRNA+ALD group; red arrows show cells with brown-stained cytoplasm but blue-stained in nuclei, two of these cells was amplified at the upper right corner of images of p.control.shRNA+ALD and ALD groups. (b) Quantification of TUNEL staining (n = 20). (c) Immunochemistry staining of Ki67 and α-SMA (400×magnification); black arrows show cells with brown-stained in cytoplasm but blue stain in nuclei, one of these cells was amplified at the upper right corner of image of p.DDR2.shRNA+ALD group; red arrows show positive cells with both brown-stained cytoplasm and nuclei, two of these cells was amplified at the upper right corner of images of p.control.shRNA+ALD and ALD groups. (d) Quantification of immunochemical staining for Ki67 (n = 20). Data are mean ± SD. **P<0.01.

There were three kinds of cells in images of double staining for tunnel and α-SMA or ki67 and α-SMA, one is cells with brown stain in both cytoplasm and nuclei which were apoptotic or proliferate HSCs; one is cells with brown stain in cytoplasm but blue in nuclei, which were activated but not apoptotic or proliferate HSCs; the last is cells with brown stain in nuclei only which are other apoptotic cells. Our quantification chart showed the result of the ratio of apoptotic or proliferate HSCs to all activated HSCs. TUNEL staining revealed a higher ratio of dural stained cells to activated HSCs in the DDR2 shRNA than ALD group, and the ratio was higher in the ALD than normal control group (**Fig. 5a,b**). Immunohistochemistry analysis of ki67 level showed that the ratio of dural-stained cells to activated HSCs was lower for with DDR2 shRNA than ALD treatment but still higher than the normal control group (**Fig. 5c,d**). The ratio did not differ between normal control and sham groups or ALD and p.control.shRNA+ALD groups.

### Acetaldehyde Increased and DDR2 shRNA Ameliorated Liver Injury and Collagen Deposition in Rats

**Figure 6:**
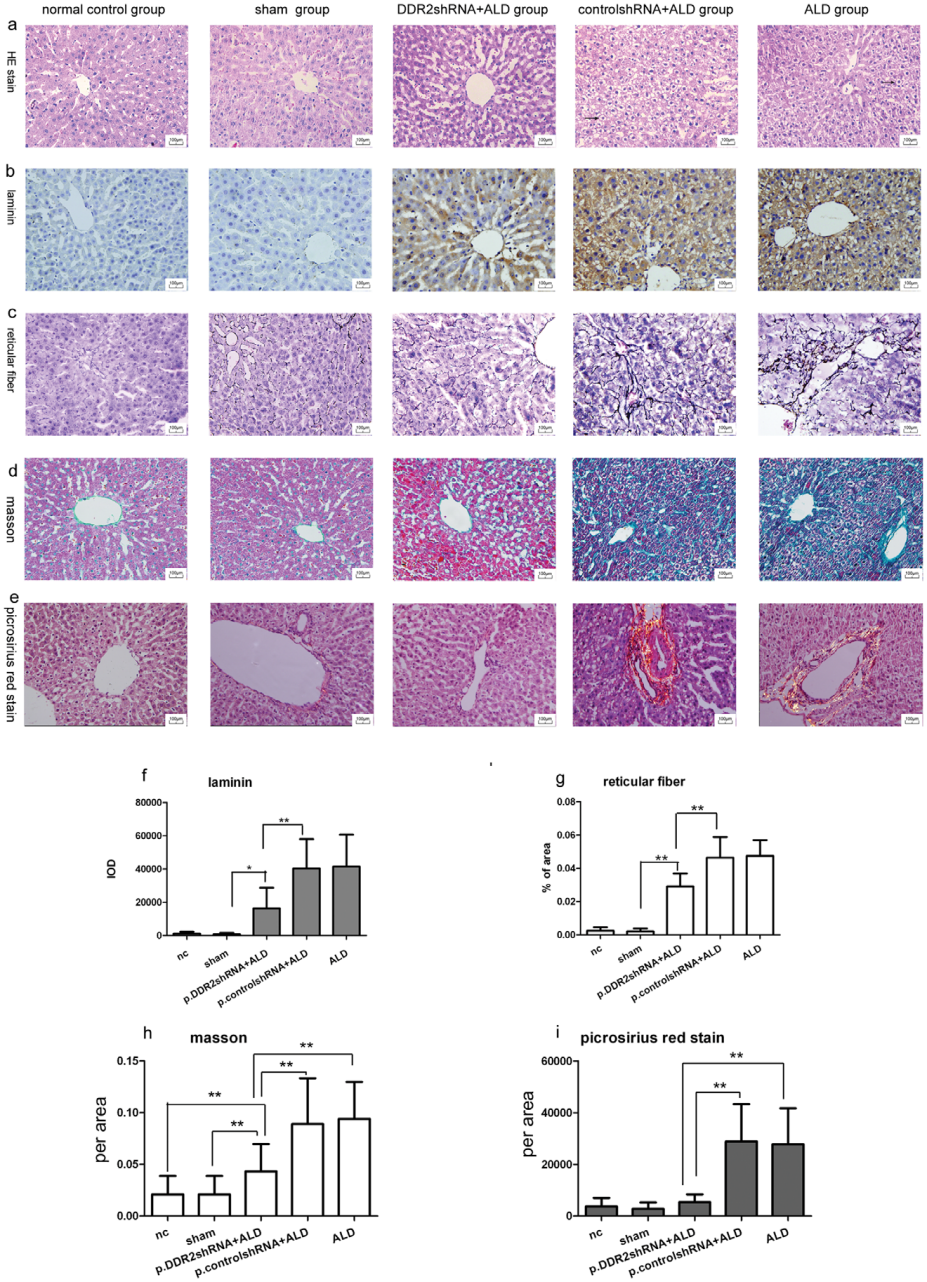
Knockdown of DDR2 inhibited injury and collagen deposition in rat liver. (a) Light microscopy of hematoxylin and eosin staining; arrows show the ballooning degeneration of hepatocytes; (b) immunohistochemistry of level of laminin; (c) Gordon and Sweet’s reticulin staining; (d) Masson staining of liver tissue; and (e) polarized light microscopy of Picrosirius-red staining (all 400×magnification). (f) Ratio of area of reticular fiber to total area. (g–i) Quantification analysis of immunohistochemistry results. Data are mean ± SD (n = 20). **P < 0.01.

Histology revealed damage in rat livers with ALD, showing ballooning degeneration of hepatocytes, sinusoidal capilatrisation and collagen deposition. H&E staining showed swelled hepatocytes as well as enlarged, rounded and reticulated cytoplasm. Chronic inflammatory cells infiltrated liver tissue (**Fig. 6a**). Reticular fiber staining and immunostaining for laminin showed greater deposition of ECM for ALD than normal control group (**Fig. 6b, c, f and g**). p.DDR2.shRNA+ALD group showed little hepatocyte ballooning degeneration and fibrous tissue proliferation. High-density interstitial matrix-enriched laminin induced by alcohol was significantly ameliorated with p.DDR2.shRNA. As well, reticular fibers and basement membrane components in a patchy sinusoidal distribution were also reduced by p.DDR2.shRNA treatment (**Fig. 6a–c,f,g**). Findings did not differ between normal control and sham groups or ALD and p.control.shRNA+ALD groups.

Acetaldehyde aggravated but DDR2.shRNA reduced collagen deposition in rat livers as compared with the normal control. Masson staining showed only a small amount of collagen located in the sinusoid wall around the central veins in the normal group but excess collagen deposited in fibrous tissue in the ALD group (**Fig. 6d,h** **)**. Picrosirius-red staining for collagen showed similar results (**Fig. 6e,i**). Intrahepatic collagen accumulation was a common feature in normal rats, but a large amount of collagen deposited along liver blood vessels in the ALD group. p.DDR2.shRNA reversed collagen deposition as compared with the ALD group. Collagen deposition did not differ between normal control and sham groups or ALD and p.control.shRNA+ALD groups.

**Figure 7:**
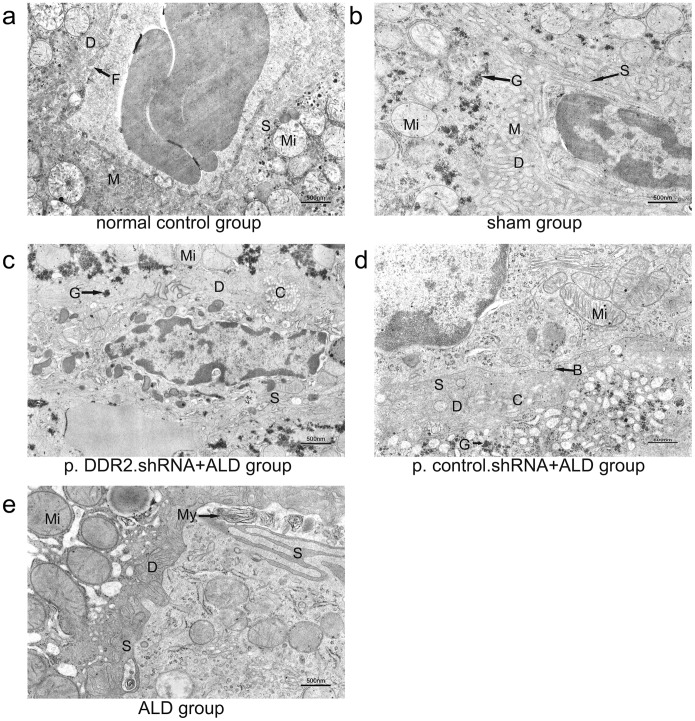
Ultrastructure of liver in rat groups. (a) Control group. (b) Sham group. (c) p.DDR2.shRNA+ALD group. (d) p.control.shRNA+ALD group. (e) ALD group. B: basement membrane. C: collagen fibers. D: Disse space. F: fenestra. G: glycogen particles. M: microvilli. Mi: mitochondria. My: myelin figure. S: Sinusoidal endothelial cells.

Electron microscopy revealed normal liver ultrastructure in control rat livers: sinusoidal endothelial cells (SECs) were thin, with a few fonestrae between them. The Disse space was filled with coarse microvilli in parenchymal cells, and the boundary of the Disse space was unclear (**Fig. 7a**). The liver ultrastructure of the normal control and sham groups was similar in terms of SECs and the Disse space; the cell matrix had many petal-shaped glycogen granules (**Fig. 7b**). The liver ultrastructure of the p.DDR2.shRNA+ALD group showed thin SECs, with fenestrae between cells. Microvilli could be seen in the Disse space. Glycogen granules were enlarged (**Fig. 7c**). The liver ultrastructure of the p.control.shRNA+ALD group showed flattened and irregular SECs, with few fenestrae. Few microvilli could be seen in the Disse space. The basement membrane started to form (**Fig. 7d**). ALD treatment produced elongated SECs, with few fenestrae between them. Cytoplasmic mitochondria were swelled and irregular, whereas cristae became vague and lost. Myelin figures could be seen (**Fig. 7e**).

## Discussion

We investigated the role of DDR2 in HSCs and rats with ALD by DDR2 shRNA knockdown. Alcohol-induced upregulation of DDR2 *in vitro* and *in vivo* was associated with upregulated expression of MMP2, collagen deposition and ECM remodeling. We established a p.DDR2.shRNA plasmid for transfection in HSC-T6 cells and rats with ALD, with reduced expression of DDR2 *in vitro* and *in vivo*. DDR2 shRNA inhibited HSC activation and once activated, it inhibited proliferation and induced cell death. Alcohol-induced liver injury and collagen deposition in rats was significantly improved with p.DDR2.shRNA injection. DDR2 gene silencing may be effective in preventing early-stage ALD.

ALD can be classified as a wound-healing response to chronic stimuli of acetaldehyde characterized by excess deposition of ECM proteins that disrupts the normal architecture of the liver and results in pathophysiological damage to the organ. HSCs are primary sources of ECM proteins, and activation of HSCs is responsible for increased synthesis and deposition of type I collagen [27]. With acetaldehyde stimulation, HSCs change from quiescent vitamin-A-storing cells to activated myofibroblast-like cells, which proliferate and become fibrogenic. *In vivo*, the spectrum of ALD includes alcoholic fatty liver, alcoholic steatohepatitis, alcoholic cirrhosis and increased risk of hepatocellular carcinoma [35]. Alcohol primarily targets nonparenchymal hepatic sinusoidal and perisinusoidal cells, thereby promoting sinusoidal capillarization, which impairs microcirculatory exchange of nutrients and waste and promotes tissue fibrosis [36]. *In vitro*, acetaldehyde stimulates type I collagen synthesis and gene transcription in cultured rat HSCs [37], [38]. We demonstrated that acetaldehyde could induce HSC activation and proliferation, cause liver injury and collagen deposition, and change body features. Thus, inhibiting HSC activation and proliferation or promoting HSC necrosis and apoptosis can reduce ECM and collagen synthesis, thereby delay or reverse the process of ALD.

Discoidin-related tyrosine kinases are novel collagen receptors expressed in mesenchymal cells that can control cellular responses to the ECM. DDR2 can be activated by collagen, which combines with a specific DDR2-DS domain mediated by overexpression of MMP2. As well, DDR2 induces MMP2 expression and provokes collagen I synthesis, MMP2 and type I collagen are directly related to the activation of HSCs during liver injury in a positive feedback loop [39], [40]. However, some experiments *in vitro* demonstrated that DDR2 inhibits fibrillogenesis of collagen type I and attenuates chronic hepatic fibrosis. As well, inhibition of MMP2 can amplify chronic liver fibrosis [18], [29], [41], [42]. So we supposed that DDR2 may play a different role in certain stages of chronic liver disease. Because the ingestion course of alcoholic liver fibrosis model is from 12 to 20 weeks [23], [43], we tried to treat rats with alcohol for 10 weeks. As a result, acetaldehyde or alcohol induced the expression of DDR2 *in vitro* and *in vivo*. Cell activation and proliferation were lower; necrosis rate and apoptosis rate were higher for HSCs with DDR2 inhibition than with acetaldehyde treatment. MMP2 expression was associated with DDR2 expression *in vitro* and *vivo*. Liver injury, collagen deposition and body features were associated with DDR2 expression in the early stage of ALD. Furthermore, the expression of the TGF-β1 signaling pathway including the expression of TGF-β1 and p-smad2/3 [44], [45], TIMP1 and collagen I, markers of liver injury [46], were reduced with DDR2shRNA treatment in HSC-T6 cells and rat liver. Thus, DDR2 could play a positive role in aggravating early-stage ALD, and silencing DDR2 could effectively prevent early-stage ALD.

There are some problems to be solved. First, we did not use primary cultured stellate cells *in vitro* because the culture and transfection of cells would last for more than 2 weeks; a stable cell line was needed, and primary cultured stellate cells would not meet our demand. In addition, many researchers have used HSC-T6 cells in lieu of rat primary HSCs [47], [48], [49]. Second, because HSCs are located in perisinusoidal spaces and DDR2-positive cells were found in the same area, DDR2 was detected only in activated HSCs but not in hepatocytes or Kupffer cells [22]. Immunohistochemical staining revealed that DDR2-positive cells were HSCs. Third, the body weight, health conditions and liver injury of rats have significant differnences between gene-therapy and normal groups. For example, the ALT level between p.DDR2.shRNA+ALD group and ALD group had no significant differences, which may be because an elevated ALT level is considered a consequence of hepatocyte damage [50], however, DDR2 correlated HSCs activation and collagen deposition, so we suspected that DDR2 could not directly reduce the injury from alcohol to hepatocytes, once the ALT level elevated which was induced by 2-week alcohol ingestion, it would not decline rapidly through DDR2 knockdown treatment. Alcohol is toxic to hepatocytes, causes inflammation in liver, and irritates the digestive system [51]. As a result, gene-therapy group’s final weight had differences compared to normal group. Fourth, although intravenous injection of a large volume of plasmids can achieve effective localization mainly in the liver [31], we could not completely reverse the damage, perhaps because DDR2 could not be silenced completely, and many other cytokines participate in ALD. Meanwhile, our research mainly focused on the effect of gene therapy with ALD, but the side effects of DDR2 knockdown in other DDR2-expressing organs needs to be examined. Fifth, as the disease develops, the effect of DDR2 along with MMP2 may be more prominent in disrupting collagen than activating HSCs, so DDR2 may play different roles in the early stage of ALD and in the fibrosis stage of chronic liver disease, which has been demonstrated in a CCl_4_-induced liver fibrosis model [30], [52]. Indeed, MMP2 level is correlated with DDR2 level, and MMP2 has differential effects on HSCs being proliferative or apoptotic depending on its concentration. During the early stage of liver injury, although MMP2 is induced by DDR2, MMP2 activity is still restrained by increased TIMP expression. Limited collagen degradation by MMP2 contributes to expanded HSC numbers, and MMP2 can induce HSC activation and proliferation [24]. During the resolution of fibrosis, when TIMP expression decreases, unrestrained MMP2 degradation of the matrix might contribute to apoptosis of HSCs [52], [53]. So DDR2, via MMP2, plays an important and controversial role in different stages of ALD.

In conclusion, we established a p.DDR2.shRNA knockdown plasmid for transfection into HSC-T6 cells and rats with ALD. Inhibition of DDR2 could inhibit HSC proliferation, induce HSC death, and ameliorate liver injury and collagen deposition in rats with early-stage ALD. As well, DDR2 was closely associated with MMP2 expression. DDR2 may play an important and controversial role in the pathogenesis of ALD along with MMP2. As a therapeutic target for managing early-stage ALD, DDR2 warrants further study.

## Acknowlegments
